# Single-Cell RNA Sequencing Reveals Alterations in Patient Immune Cells with Pulmonary Long COVID-19 Complications

**DOI:** 10.3390/cimb46010029

**Published:** 2024-01-02

**Authors:** Kristīne Vaivode, Rihards Saksis, Helēna Daiga Litvina, Helvijs Niedra, Marta Līva Spriņģe, Una Krūmiņa, Jānis Kloviņš, Vita Rovite

**Affiliations:** Latvian Biomedical Research and Study Centre, LV-1067 Riga, Latviamarta.springe@biomed.lu.lv (M.L.S.);

**Keywords:** long COVID-19, neutrophils, CD8+ NKT cells, scRNA-seq, ScType

## Abstract

Since the emergence of the COVID-19 pandemic, the effects of SARS-CoV-2 have been extensively researched. While much is already known about the acute phase of the infection, increasing attention has turned to the prolonged symptoms experienced by a subset of individuals, commonly referred to as long COVID-19 patients. This study aims to delve deeper into the immune landscape of patients with prolonged symptoms by implementing single-cell mRNA analysis. A 71-year-old COVID-19 patient presenting with persistent viral pneumonia was recruited, and peripheral blood samples were taken at 3 and 2 years post-acute infection onset. Patients and control peripheral blood mononuclear cells (PBMCs) were isolated and single-cell sequenced. Immune cell population identification was carried out using the ScType script. Three months post-COVID-19 patients’ PBMCs contained a significantly larger immature neutrophil population compared to 2-year and control samples. However, the neutrophil balance shifted towards a more mature profile after 18 months. In addition, a notable increase in the CD8+ NKT-like cells could be observed in the 3-month patient sample as compared to the later one and control. The subsequent change in these cell populations over time may be an indicator of an ongoing failure to clear the SARS-CoV-2 infection and, thus, lead to chronic COVID-19 complications.

## 1. Introduction

The coronavirus disease (COVID-19) pandemic, caused by the severe acute respiratory syndrome coronavirus 2 (SARS-CoV-2), emerged in late 2019 and has since impacted millions of lives globally. While the acute phase of the infection has been extensively studied and characterized, increasing attention has turned to the prolonged symptoms experienced by a subset of individuals, commonly referred to as “Long COVID-19” or “Post-Acute Sequelae of SARS-CoV-2 Infection” [1].

Long COVID-19 presents with a broad spectrum of symptoms, ranging from persistent fatigue, dyspnea, and chest pain to neurological manifestations like cognitive dysfunction, anosmia, and mood disturbances [2]. These symptoms can persist for months after the acute phase of the infection has resolved and, in some cases, can significantly impair daily functioning and quality of life.

The exact prevalence of long COVID-19 is still being elucidated, but studies have indicated that a substantial proportion of COVID-19 survivors may experience at least one prolonged symptom [3]. Risk factors for developing long-term COVID-19 are still being defined, but evidence suggests that factors such as disease severity, age, and certain comorbidities may influence the likelihood of experiencing these prolonged effects [4].

Neutrophils, one of the most abundant types of white blood cells, play a crucial role in the body’s innate immune response. Recent studies suggest that these cells may be pivotal in the progression and severity of COVID-19 infections. Elevated neutrophil counts, often seen in COVID-19 patients, have been correlated with disease severity, increased inflammation, and poorer outcomes [5,6,7]. These cells release neutrophil extracellular traps (NETs), which, although designed to ensnare pathogens, can cause damage to host tissues and contribute to the hyperinflammation observed in severe cases of COVID-19. Also, the severity of the disease can be linked to the overproduction of pro-inflammatory factors that in turn can lead to various organ injuries and even failure [8]. Nonetheless, the exact role of neutrophil populations in such severe long COVID-19 patients still remains to be elucidated.

Natural killer T (NKT) cells are a subset of T cells that share characteristics with both T cells and natural killer (NK) cells. They play a role in bridging innate and adaptive immunity. While there is an understanding of a decline in NKT cells in severe acute COVID-19 cases [9], more research is needed to clarify their exact role and changes in the post-acute or chronic phases of the disease.

Single-cell RNA sequencing (scRNA-seq) is a powerful tool that enables the analysis of the whole transcriptome within each cell individually [8]. The data obtained from scRNA-seq allows us to quantify and represent the expression of genes present within the individual cells of a sample, such as mononuclear cells from the peripheral blood samples of patients [10]. This allows the researchers to identify different populations, including rare subsets of immune cells, and track the immune cell composition and cell type-specific gene expression changes in the presence of a disease [11,12]. Integrating scRNA-seq data with the information about the pathology and symptomatic observations of patients provides insight into the influence of the genetic profile of a population of individual cells on the activated molecular mechanisms and allows further investigation of the immune response within an acute or chronic condition [13].

Several scRNA-seq studies have already investigated the immunological landscape alterations in PBMCs of COVID-19 patients, showing activation of monocytes with strong upregulation of antiviral INF response genes [10,14,15,16]. Furthermore, a study by Shrepping et al. observed that the immunological profile of severe disease cases in comparison to mild cases consists of HLA-DR^low^ dysfunctional monocytes with strong upregulation of genes associated with pro-inflammatory macrophages, followed by upregulation of immature neutrophils [16]. Despite this, the immune cell composition and altered gene expression profiles remain to be explored in long COVID-19 patients using the scRNA-seq approach.

Understanding the pathophysiology, clinical manifestations, and potential interventions for long COVID-19 is paramount in our ongoing efforts to combat the broader repercussions of the pandemic. This publication aims to consolidate the existing knowledge on long COVID-19 by showing that partially neutrophil and CD8+ NKT-like cells are affected in long COVID-19 patients’ blood as compared to healthy persons. Understanding the role of these cell types in long COVID-19 may provide insights into its pathophysiology and potential therapeutic strategies. For instance, if persistent activation of these cells contributes to ongoing symptoms, treatments targeting their stabilization or modulation may offer relief.

## 2. Materials and Methods

### 2.1. Sample Collection

Long COVID-19 patent (age 71, BMI 23.9) peripheral blood samples were collected at Paula Stradins Clinical University Hospital (PSCUS) 3 and 2 years post-acute COVID-19 onset. The patient had pulmonary complications in the form of viral pneumonia that lasted for several months after the acute COVID-19 infection. Peripheral blood samples were collected in BD Vacutainer^®^ CPT™ and kept at room temperature. Control was a 27-year-old female without any chronic conditions. Control and patient samples were processed according to the manufacturer’s instructions up to 2 h post collection. In brief, vacutainers were centrifuged at 1800 RCF for 20 min at room temperature. The PBMC layer was then collected, washed with RPMI medium, and frozen in fetal bovine serum (FBS) with added 10% DMSO. PBMCs were stored in liquid nitrogen until further processing.

### 2.2. RNA Library Preparation

PBMC cell suspension was prepared using Phosphate-buffered Saline (PBS) with added 0.04% Bovine Serum Albumin (BSA, Sigma-Aldrich A-2153, St. Louis, MO, USA) for further processing by DNBelab C Series High-throughput Single-cell RNA Library Preparation Set V2.0 (MGI, Shenzhen, China). Live cells were counted, indexed, and formed into single-cell-containing oil droplets using DNBelab C4 station, followed by mRNA capture using magnetic beads and demulsification. mRNA was further reverse transcribed and purified, and cDNA, along with oligo libraries, were constructed according to the manufacturer’s instructions.

Quality control was performed on cDNA and Oligo products, as well as cDNA and Oligo libraries using Qubit^®^ 2.0 Fluorometer (Thermo Fisher Scientific, Waltham, MA, USA) and the Qubit dsDNA HS Assay kit (Thermo Fisher Scientific, Waltham, MA, USA) for concentration measurement and Agilent 2100 Bioanalyzer System (Agilent Technologies, Santa Clara, CA, USA) for fragment size distribution.

### 2.3. Sequencing

DNB (DNA Nano Ball) generation was performed with 10 ng circularization ssDNA libraries (cDNA library and Oligo library) using DNBSEQ-G400RS High-throughput Sequencing kit FCL PE100 (MGI, Shenzhen, China) according to the manufacturer’s protocol, respectively. The reaction is at 30 °C, 30 min for cDNA library, and at 30 °C, 20 min for oligo library. Finally, the DNBs were sequenced on the DNBSEQ-G400RS platform (MGI, Shenzhen, China) with 30 bp read1 and 100 bp read2 for cDNA, 20 bp for read1 and 30 bp read2 for Oligo.

### 2.4. Preliminary Data Analysis

Preliminary data analysis was performed using the DNBelab C Series HT scRNA analysis workflow, which performs sequencing read alignment using STAR (GRCh38) while using Sambamba and PISA for aligned file manipulation, filtering and feature counting. Reads with a PHRED score of less than 20 along with mapping quality less than 20 were filtered. UMI correction was performed using the Hamming distance and frequency by retaining only those UMIs with the highest counts.

### 2.5. Single-Cell RNA Sequencing Data Analysis Using Seurat v5 R Package

Filtered gene expression matrices (including cell barcodes, gene list and matrix itself) were uploaded in R (version 4.3.1) and created as Seurat objects. Data were then filtered retaining cells with unique feature counts (genes) over 5000 or less than 500. Data were then log normalized with scale factor 10,000 and the top 2000 variable features were identified. Immune system-related anchors were identified and the three datasets were integrated. The data was then scaled and linear dimension reduction was performed using principal component (PCA) analysis and uniform manifold approximation and projection (UMAP) for visualization.

### 2.6. Cell Type Identification Using ScType R Package

Instead of manually assigning cell populations, we used a fully automated cell type identification open-source R-package ScType [17] based solely on a given scRNA-seq data, along with a comprehensive cell marker database as background information. The unbiased yet selective cell-type annotation is achieved by compiling the largest database of established cell-specific markers (ScType database) and by ensuring the specificity of marker genes across both the cell clusters and cell types.

## 3. Results

### 3.1. Comprehensive Single-Sell Transcriptomic Analysis and Automated Sell-Type Annotation in Long COVID-19 PBMCs

**Figure 1:**
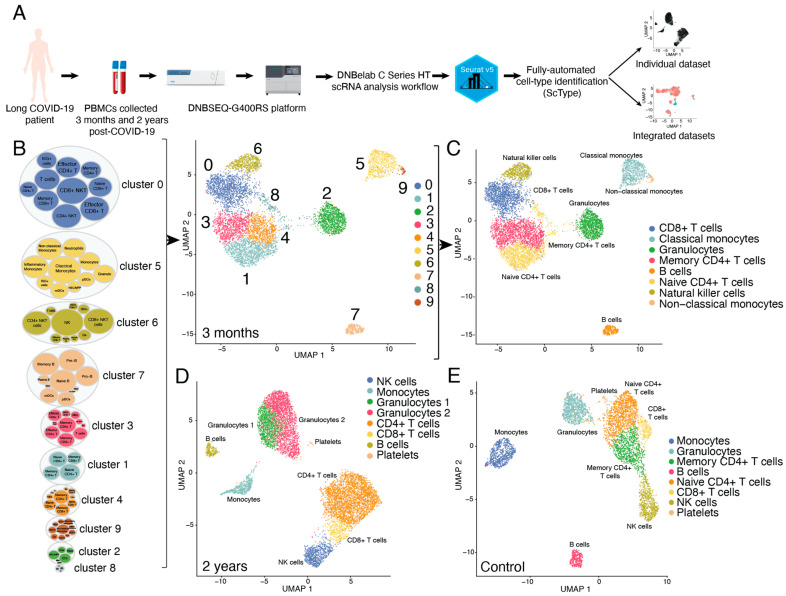
Automated immune cell identification using ScType. (**A**) Pipeline for long COVID-19 and control blood sample processing and analysis. (**B**) UMAP visualization of scRNA-seq profile of 9106 PBMCs for 3 months post-acute COVID-19 patient sample and ScType immune cell population likelihood bubble plots (summarized in Figure S1). (**C**) UMAP visualization of ScType immune cell profile of 9106 PBMCs for 3 months post-acute COVID-19 patient sample. (**D**) UMAP visualization of ScType immune cell profile of 10,526 PBMCs for 2-year post-acute COVID-19 patient sample. (**E**) UMAP visualization of ScType immune cell profile of 7510 PBMCs for the control sample.

Pulmonary long COVID-19 patient’s PBMCs were collected and isolated three months and two years post-acute COVID-19 infection and single-cell transcriptomes were sequenced using the DNBSEQ-G400RS platform (sample analysis workflow is depicted in Figure 1A). To analyze and visualize the results, we used Seurat (version 5.0.0) and then ScType script. ScType is a fully automated cell-type identification based on a given scRNA-seq data and a comprehensive cell marker database as background information taken from six scRNA-seq datasets [17]. Initially, we analyzed each dataset separately, starting with the long COVID-19 patients sample collected three months post-acute infection (Figure 1B and Figure S1). Seurat pipeline with resolution 0.8 produced 10 clusters; however, the ScType algorithm identified clusters 3 and 4 as both giving the strongest signal for memory CD4+ T cells. Cell population assignment potential is represented in the bubble plot showing all the cell types that ScType considered for cluster annotation. The outer bubbles correspond to each cluster, where the bigger the bubble, the more cells there are, while the inner bubbles correspond to considered cell types for each cluster. This analysis allowed comprehensive cell type annotation, including all main immune cell populations (Figure 1C). Interestingly, in all three datasets, granulocyte populations were identified as usually in single-cell analysis, granulocytes generally remain a significant challenge (Figure 1C–E). In addition, we could identify CD4+ and CD8+ T cells, natural killer (NK) cells, B cells, and monocytes.

### 3.2. Dynamics of Neutrophil Populations and CD8+ NKT-Like Cells in Long COVID-19 Patient vs. Control

**Figure 2:**
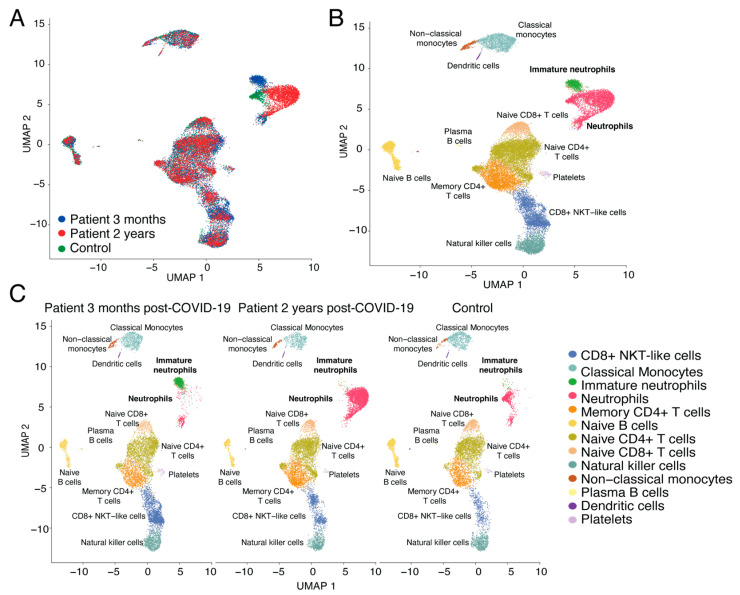
Integrated dataset analysis results. (**A**) UMAP visualization of scRNA-seq profile of 27,142 PBMCs for integrated 3-month (blue), 2-year-long COVID-19 (green), and control (red) samples. (**B**) UMAP visualization of ScType immune cell profile for the integrated dataset. (**C**) UMAP visualization of ScType immune cell profile for separate 3-month, 2-year-long COVID-19 and control samples.

Next, we integrated the three datasets to visualize the immune cell population differences in long COVID-19 patients and the control samples (Figure 2A). We could immediately see that there were differences in one area of the UMAP. When identifying the potential immune cluster identities using ScType, it became apparent that the contrasts were in the granulocyte populations, namely neutrophils (Figure 2B). When separating the three datasets, we could see that three months after the acute infection, the long COVID-19 patients sample had a prominent immature neutrophil population in their PBMCs, which had expressed markers such as CD24 and DEFA3 (Figure 2C), and this population had almost diminished after 2 years. Furthermore, the control sample had very few immature neutrophils. In contrast, it did have a substantial mature neutrophil population, which was also present in the long COVID-19 patients’ two-year sample, and the patient’s blood in this neutrophil population was much more extensive (Figure 2C). In addition, notable changes could be observed in the CD8+ NKT-like cells with the highest numbers present in the three-month patient sample, diminished numbers at two years post-acute disease, and the lowest in the control sample.

## 4. Discussion

The intricate relationship between immune responses and long COVID-19 continues to be a topic of significant interest, with scRNA-seq providing a window into the cellular dynamics at play during different stages of infection and recovery. The results obtained from the analysis of PBMCs from long COVID-19 patients at distinct time points highlight several findings that could provide deeper insights into the immune system’s prolonged response and the potential therapeutic targets.

Neutrophils are the most abundant white blood cells and frontline defenders of our innate immune system [18]. Their increased presence in the PBMCs of patients, particularly two years post-acute COVID-19 infection, indicates a sustained inflammatory response. Traditionally, granulocytes, especially neutrophils, have been challenging to identify in single-cell analysis, primarily due to their short lifespan and rapid turnover. However, identifying granulocyte populations in all three datasets underscores the implemented analysis pipeline’s efficiency and emphasizes their potential significance in the immune landscape of long COVID-19 patients. The prominence of immature neutrophils three months post-acute infection suggests an ongoing active immune response, possibly pointing towards a delay in the maturation of these cells. The transition from a higher immature to mature neutrophil gene expression profile in the two-year post-infection sample might indicate a shift toward immunological stabilization. This contrasts sharply with control samples, where mature neutrophils dominate, suggesting a standard, non-inflammatory state. These results also coincide with results obtained by Shrepping et al., where they show that severe COVID-19 is marked by the upregulation of neutrophil precursors and immature neutrophils [16].

The observed changes in CD8+ NKT-like cell populations add another layer of complexity. Their high numbers in the three-month patient samples imply a heightened adaptive immune response, possibly to combat the viral remnants or mitigate the dysregulated inflammatory processes. The subsequent decline in these populations over time, reaching their lowest in the control samples, may indicate either a return to baseline post infection or the potential exhaustion of these cells due to prolonged activation. Further research is imperative to distinguish between these possibilities.

Nonetheless, the functional roles of these altered cell populations remain to be determined. Investigations into the functional capabilities of the immature neutrophils, or the cytokine profiles and cytotoxic activities of the CD8+ NKT-like cells from long COVID-19 patients compared to controls, could provide actionable insights.

### Limitations of Study

The primary limitation of the study is the inclusion of only one patient with pulmonary long COVID-19 complications. This limited sample size can reduce the generalizability of the findings to the broader population of individuals with long COVID-19. Moreover, the study captures data at just two time points post-acute COVID-19 infection, and given that long COVID-19 may have evolving characteristics, more frequent observations might have offered a more comprehensive understanding of the progression of immune responses. While the study utilized advanced single-cell analysis techniques, inherent limitations of these methods, such as cell viability, dropout events and sequencing depth, could have impacted the results. It is also worth noting that every individual’s immune response can be unique. By focusing on PBMCs from a singular patient, there is a risk that the study captured idiosyncratic immune responses rather than representative changes associated with long COVID-19. It should be taken into consideration that the severity and mortality in male COVID-19 patients are higher than in females; thus, a sex-diverse cohort analysis would be beneficial [19,20]. While the study did make comparisons with control samples, the inclusion of a more diverse control group with varied health statuses, ages and other demographic factors might have added value. In the current study, we were able to include a control sample of relatively young females without any chronic conditions, which helps to acknowledge our data with the healthy status of blood immune cells, as indicated above we recognize the need for furthering data to other controls as well. We clearly see the tendencies in our data that align with the literature about neutrophil functionality and fluctuations. Furthermore, our study is the first to show long-COVID-19 consequences on immune cells at a single-cell level of the same patient in a longitudinal manner, which merits the significance of the obtained results. The study’s emphasis was primarily on transcriptomic changes. The addition of functional assays could have shed light on the actual capabilities and roles of the identified immune cell populations in the pathogenesis of long COVID-19.

## 5. Conclusions

In summary, single-cell RNA sequencing has provided an unparalleled view into the cellular landscapes of long COVID-19 patients at different stages post infection. The distinct immune signatures observed across different time points and in comparison to controls emphasize the dynamic nature of the immune response to SARS-CoV-2. Future studies should focus on elucidating the functional implications of these cellular changes and their potential roles in driving or mitigating the long-term symptoms associated with COVID-19.

## Data Availability

Data are currently being submitted to a publicly accessible repository and will be available after the reviewing process.

## Supplementary Materials

The following supporting information can be downloaded at: https://www.mdpi.com/article/10.3390/cimb46010029/s1, Figure S1: ScType bubble plot results presented by the cell type likelihood in a receding order.
