# Supplementary material for: Single-Cell RNA Sequencing Reveals Alterations in Patient Immune Cells with Pulmonary Long COVID-19 Complications
Source: Curr Issues Mol Biol. 2024 Jan 2;46(1):461–8. doi: 10.3390/cimb46010029 (PMC10814809; doi:10.3390/cimb46010029)
Supplement: Supplementary file 1 [file cimb-46-00029-s001.zip › cimb-2749454-supplementary.pdf]

Supplementary figure S1 for Fig. 1.

Cluster	Colour	Bubbles in receding order									
0		CD8+ NKT	Effector CD8+ T	CD4+ NKT	T cells	Memory CD8+ T	Naive CD8+ T	ISG+ cells	Memory CD4+ T	Naive CD4+ T	
5		Classical Monocytes	Inf. Monocytes	Non-classsical monocytes	Neutrophils	Monocytes	Granulocytes	mDCs	pDCs	ISG+ cells	HPS/MPP
6		NK	CD8+ NKT	CD4+ NKT	Granulo.	ISG+ cells	T cells	Effector CD8+ T	Naive CD8+ T	Plasma B	Non-callical mono
7		Memory B	Naive B	Pre-B	Pro-B	mDCs	pDCs	Plasma B	Mast cells	Monocytes	HPS/MPP
3		Memory CD4+ T	Memory CD8+ T	Effector CD4+ T	Effector CD8+ T	T cells	Megakarocytes	HPS/MPP	Eosinophils	Naive CD8+ T	
1		Naive CD4+ T	Memory CD4+ T	Naive CD8+ T	Memory CD8+ T						
4		Memory CD4+ T	Memory CD8+ T	Naive CD4+ T	Effector CD4+ T	Megakarocytes	T cells	Cancer	HPS/MPP		
7		Non-classical Mono	Monocytes	Inf. Monocytes	Granulocytes	mDCs	pDCs	Neutrophils	ISG+ cells	Classical Monocytes	
2		Eosinophils	Granulocytes	HPS/MPP	Basophils						
8		Naive CD4+ T	Naive CD8+ T	Cancer	Effector CD4+ T						

Figure S1. ScType bubble plot results presented by the cell type likelihood in a receding order (corresponding to Fig. 1B).
